# New paradigms in cervical cancer prevention: opportunities and risks

**DOI:** 10.1186/1472-6874-8-23

**Published:** 2008-12-17

**Authors:** Guglielmo Ronco, Paolo Giorgi Rossi

**Affiliations:** 1 Unità di Epidemiologia dei Tumori, CPO Piemonte, Torino, Italy; 2 Laziosanità – Agenzia di Sanità Pubblica, Regione Lazio, Roma, Italy

## Abstract

Testing for the DNA of high-risk types of papilloma virus (HPV) is more sensitive than cytology in detecting pre-cancerous lesions. One of the main advantages will be the possibility of applying prolonged screening intervals. However adequate screening protocols (age of start and stop, screening intervals, management of HPV positive women) need to be applied in order to avoid over-referral to colposcopy and over-treatment and to maintain sustainable costs. Further follow-up of running trials and research on molecular markers will better define these parameters. The new situation will require organised screening programmes with rigorous protocols and monitoring. This will be even more needed when women vaccinated for HPV 16 and 18 will be screened. Research on how to best screen vaccinated women is a priority. This paper proposes an overview of the plausible impact of new technologies in cervical cancer screening in the near future and in the vaccinated cohorts.

## Background

### Human Papilloma virus (HPV) as primary screening test of cervical cancer precursors

Both studies based on double-testing of the same women [1,2] and large population trials [3-8] showed that testing for the DNA of high-risk HPV types is more sensitive but less specific than conventional cytology in identifying high-grade cervical intraepithelial neoplasia (CIN).

HPV testing detects almost all high-grade CINs identified by cytology [1,2]. As a result, almost the same sensitivity is obtained with HPV alone as with both cytology and HPV together as primary screening tests (i.e. if only HPV-positive or also HPV-negative women with abnormal cytology are referred to colposcopy). However, with the combined strategy, referrals to colposcopy are much more frequent and the probability that test-positive women actually have a high-grade CIN (the Positive Predictive Value, PPV) is substantially lower [3,4].

In women at least 35 years of age, in the "New Technology in Cervical Cancer" (NTCC) trial, HPV testing was found to be 63% more sensitive than cytology when all HPV-positive women are referred to colposcopy [8]. At this age loss in specificity is small: in a pooled analysis of European and North American studies based on double-testing [1], specificity was 93.3% with HPV DNA testing vs. 97.1% with cytology. However, even with HPV alone there is a remarkable loss in PPV. In the NTCC trial the relative ratio in PPV between HPV and cytology (relative PPV) was 0.67 [8].

Thus, a number of strategies to increase specificity have been or are being evaluated. The most studied strategy – that we will refer to as "cytological triage" – involves directly referring to colposcopy only HPV-positive women who also show cytological abnormalities, while the remaining HPV-positives are retested at shorter interval and referred to colposcopy only if they retest as HPV positive [9]. This approach is based on knowledge that only persistent HPV infections are relevant to carcinogenesis. Trials based on combined HPV and cytology screening but with cytological triage of HPV-positive women at least 30 (POBASCAM [6]) or 35 (Sweedscreen [7]) years of age found sensitivities about 50% higher than with cytology alone, very similar to those obtained when all HPV positive women are referred.

The persistence of lesions detected by HPV testing versus cytology is also a relevant factor. Remarkably, the two aforementioned studies [6,7] showed that HPV testing allowed an earlier detection of persistent, therefore clinically relevant, lesions. It is plausible that both age and the application of triage had a role in such a result. The observed earlier diagnosis means that the intervals for re-screening HPV-negative women can be longer. The very low detection rate of high-grade CIN at subsequent screening in previously HPV-negative women supports this choice. Longer intervals have the clear advantage of fewer screening episodes over a lifetime and may provide the opportunity of achieving higher coverage. Further follow-up of randomised trials will provide evidence on how long HPV screening intervals can be [10].

However, cytological triage requires that some women are re-tested at short intervals, which is a disadvantage because compliance rarely is complete. Other strategies, based on molecular markers, are under study. These include viral load, genotyping, testing for the RNA of the viral oncogenes E6 and E7 and testing for the over-expression of the p16-INK4A protein [11]. Recently HPV testing with a single immediate triage test for p16-INK4A over-expression – with no further recall – showed a relative sensitivity of 1.53 with virtually no increase in referrals to colposcopy (relative referral 1.08) [12].

Among younger women the situation is slightly more complicated. At this age infection is very frequent and HPV test specificity is lower. Nevertheless, among women 25–34 years of age, in the NTCC trial, HPV testing with cytological triage resulted in a relative sensitivity vs. cytology of 1.58 with only a moderate loss in PPV (relative PPV 0.78) [4]. However, a major problem at this age is over-diagnosis. Data from the NTCC study, involving direct referral of all HPV positive women, found a very large increase in sensitivity (relative sensitivity 3.50) suggesting that a large proportion of the lesions detected by this strategy at this age are regressive. Results from the follow-up of the NTCC study, currently under analysis, will provide information on the appropriateness of HPV screening at younger ages.

## Conclusion

In conclusion, available results strongly support the adoption of HPV testing for cervical screening. However, before general routine implementation, the follow-up results of running randomised trials, which will be available soon, should be considered. They will also provide evidence on some aspects that need to be better defined: age at first testing, the best screening interval, the best management of HPV positive women. In the meantime, starting large pilot (demonstration) projects seems reasonable. This will allow us to estimate the impact of the application of new technologies in practice, evaluate costs (to date the major barrier to using HPV as the primary test is the price of the kit [13]) and set up systems for quality assurance and monitoring.

In any case, the following issues must be taken into account:

- Applying longer screening intervals represents a major advantage of HPV testing. This is also needed in order to maintain sustainable costs.

- Adequate protocols need to be applied. In their absence there is a risk not only of over-referral to colposcopy but also of overtreatment (see the case of direct referral to colposcopy of younger HPV positive women), and therefore for potential harm (excisional treatment for CIN increases the risk of complications in pregnancy [14,15]).

- Validated tests with appropriate sensitivity and more importantly, specificity must be adopted

All of these factors indicate the need for organised screening programmes that actively invite women at the appropriate intervals, adopt well-defined management protocols, register all results (the application of protocols requires accurate knowledge of women's screening experience) and monitor performances.

### New scenario in the vaccine era

Two vaccines – one against the oncogenic HPV types 16 and 18 and one that also includes the non-oncogenic types 6 and 11 – proved to be highly effective in preventing HPV16/18-linked CIN2+ [16,17]. In some countries vaccination is free of charge or actively offered to adolescents [18]. There is large consensus that vaccinated women need screening [19-21], as HPV 16 and 18 are only responsible for 75% of cervical cancers. We must understand how vaccinated women should best be screened. In vaccinated women the incidence of CIN2+ is expected to be reduced by 50–60% compared to the unvaccinated but vaccination reduces the incidence of low grade CIN and ASC-US only by 20% [22]. Thus, with cytology, the detection rate will decrease much more than referrals to colposcopy. Consequently PPV will substantially decrease. However, as non-16/18 HPV types have a lower probability of neoplastic progression, the PPV of HPV testing will also decrease in vaccinated women.

Overall, this scenario represents an additional reason for shifting to HPV testing but will require even more conservative protocols, to achieve high PPV for colposcopy referral, both in order to avoid false positive histological diagnoses (that are inversely related to the PPV of referral [23]) and to maintain sustainable overall costs of cervical cancer control. The lower incidence of high-grade CIN with non-16/18 HPV types [24] – therefore the longer interval between infection and the development of such lesions – suggests the possibility of even longer screening intervals. Research on this subject is a priority. In any case, this situation will need a coordinated program for cervical cancer prevention that will be able to integrate vaccinations and screening, ensuring appropriate prevention to all women throughout their life, according to their history of immunisation and screening.

## Abbreviations

HPV: Human Papilloma Virus; NTCC: New Technology in Cervical Cancer, multicentre randomised trial; CIN: Cervical Intraepitelial Neoplasia; CIN2+: Cervical Intraepitelial Neoplasia grade 2 or more severe; PPV: Positive Predictive Value.

## Competing interests

GR received minor payment by GenProbe for participating in internal scientific advisory boards. The Agency for Public Health received a grant by Sanofi Pasteur MSD for a study on the HPV-related burden of disease in Italy. PGR received travel reimbursement to present the results of the study at two international conferences and at an internal workshop.

## Authors' contributions

GR drafted the section on HPV testing for primary screening and PGR the section on the new scenario in the vaccine era. Both authors discussed the entire text and approved the final manuscript.

## Pre-publication history

The pre-publication history for this paper can be accessed here:


